# Supplementary material for: Spatial and temporal distribution patterns of tick-borne diseases (Tick-borne Encephalitis and Lyme Borreliosis) in Germany
Source: PeerJ. 2021 Dec 13;9:e12422. doi: 10.7717/peerj.12422 (PMC8675256; doi:10.7717/peerj.12422)
Supplement: Supplemental Information 1 [file peerj-09-12422-s001.pdf]

Supplemental Files

Spatial and temporal distribution patterns of tick-borne diseases (Tick-borne Encephalitis and Lyme Borreliosis) in Germany

Sarah Cunze, Gustav Glock, Sven Klimpel

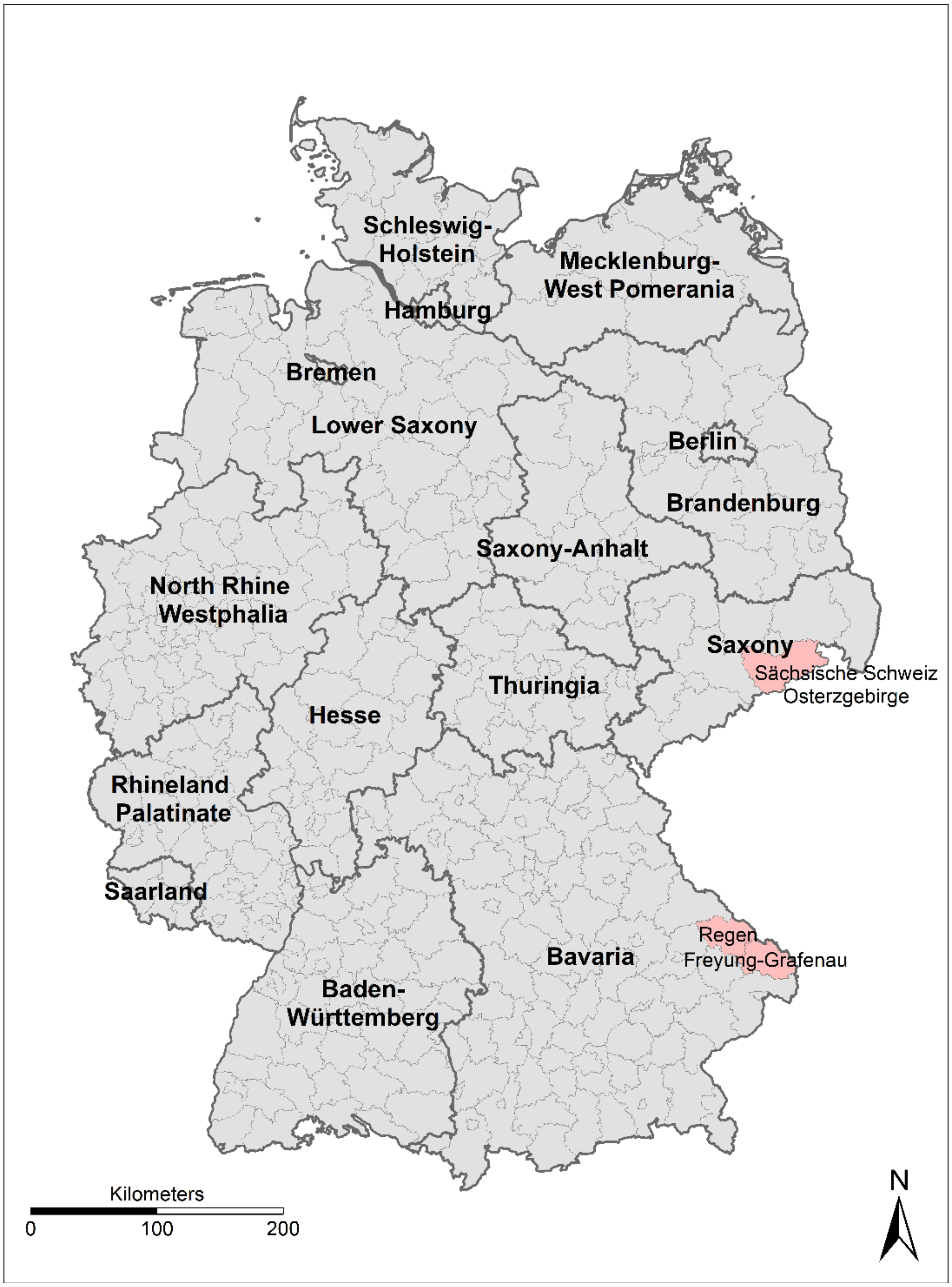

Figure S1: Federal states of Germany and certain administrative districts mentioned in the text.

Supplemental Files

Spatial and temporal distribution patterns of tick-borne diseases (Tick-borne Encephalitis and Lyme Borreliosis) in Germany

Sarah Cunze, Gustav Glock, Sven Klimpel

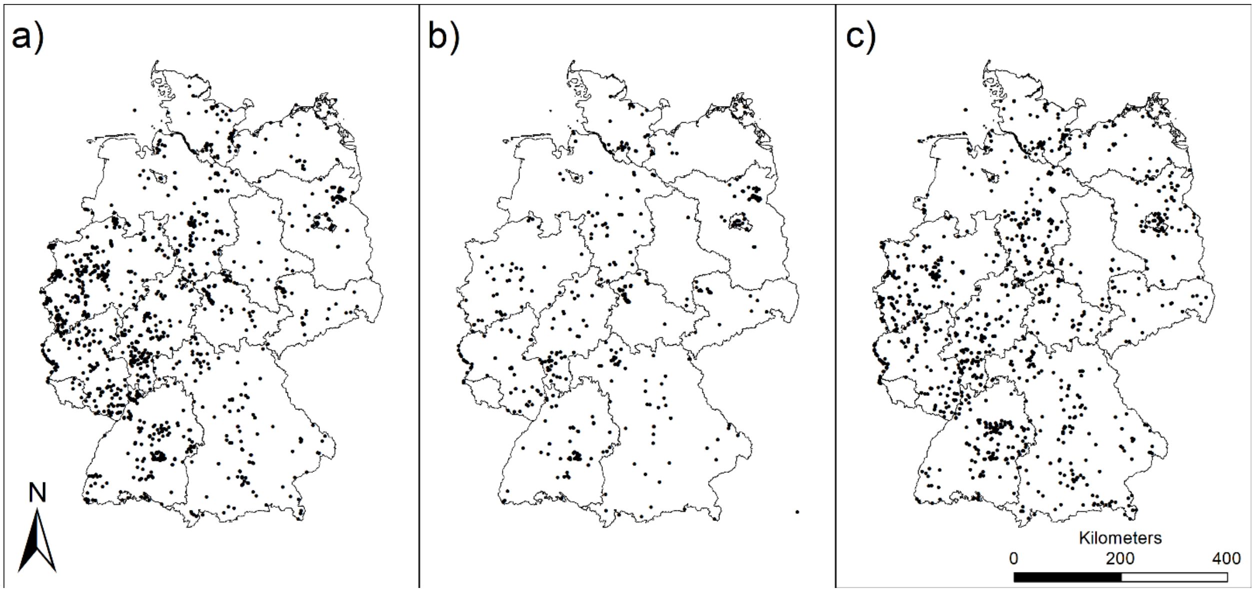

Figure S2: Distribution data for selected tick-associated small rodents in Germany serving as host for ticks in larval and nymph stages as well as reservoir hosts for TBD agents. GBIF occurrence records for a) bank vole (*Myodes glareolus* https://doi.org/10.15468/dl.82wsgb) b) yellow-necked mouse (*Apodemus flavicollis* https://doi.org/10.15468/dl.p4r693) and c) shrews (Family: Soricidae https://doi.org/10.15468/dl.czyyq2).

Supplemental Files

Spatial and temporal distribution patterns of tick-borne diseases (Tick-borne Encephalitis and Lyme Borreliosis) in Germany

Sarah Cunze, Gustav Glock, Sven Klimpel

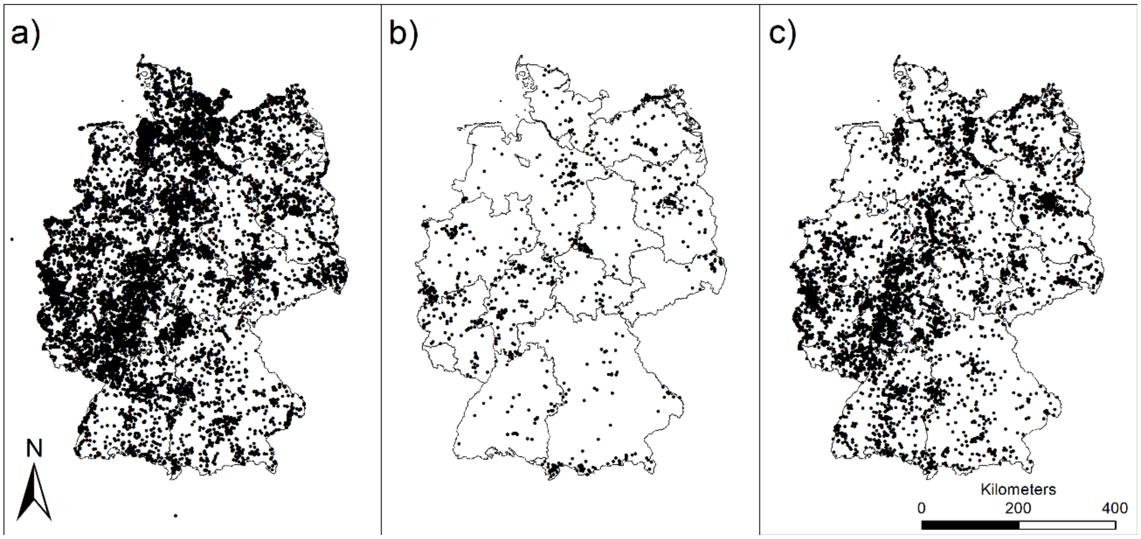

Figure S3: Distribution data for selected tick-associated game animals in Germany. GBIF occurrence records for a) roe deer (*Capreolus capreolus* https://doi.org/10.15468/dl.wp98ts) b) red deer (*Cervus elaphus* https://doi.org/10.15468/dl.72ddmz) and c) red fox (*Vulpes vulpes* https://doi.org/10.15468/dl.wgtneb).

Supplemental Files

Spatial and temporal distribution patterns of tick-borne diseases (Tick-borne Encephalitis and Lyme Borreliosis) in Germany

Sarah Cunze, Gustav Glock, Sven Klimpel

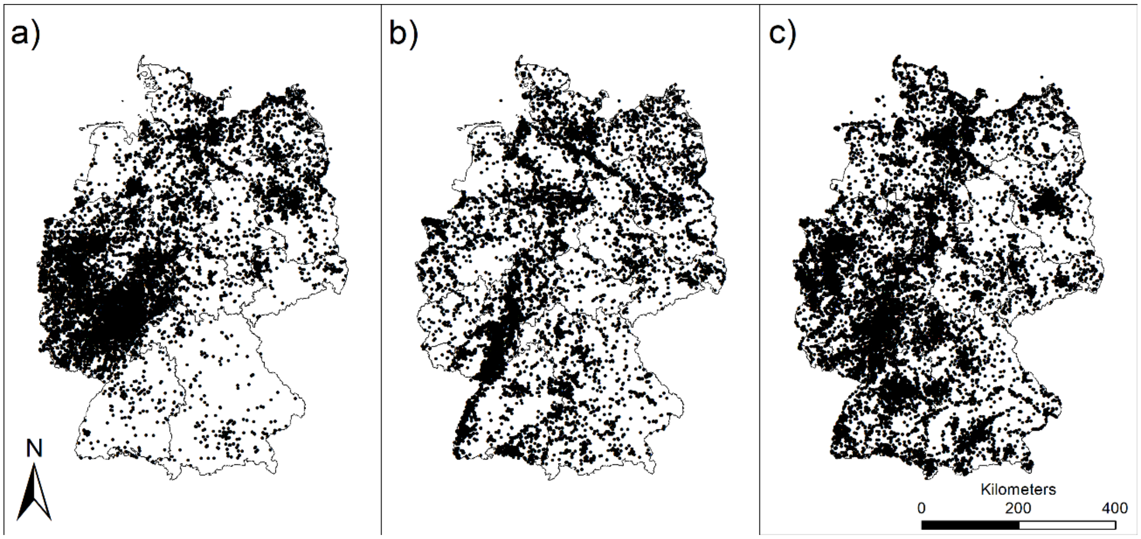

Figure S4: Distribution data for selected tick-associated bird species in Germany. GBIF occurrence records for a) Cran (*Grus grus* https://doi.org/10.15468/dl.j4fe7k) b) Stork (*Ciconia ciconia* https://doi.org/10.15468/dl.k6jv69) and c) blackbird (*Turdus merula* https://doi.org/10.15468/dl.t3h8b4).

Spatial and temporal distribution patterns of tick-borne diseases (Tick-borne Encephalitis and Lyme Borreliosis) in Germany

Sarah Cunze, Gustav Glock, Sven Klimpel

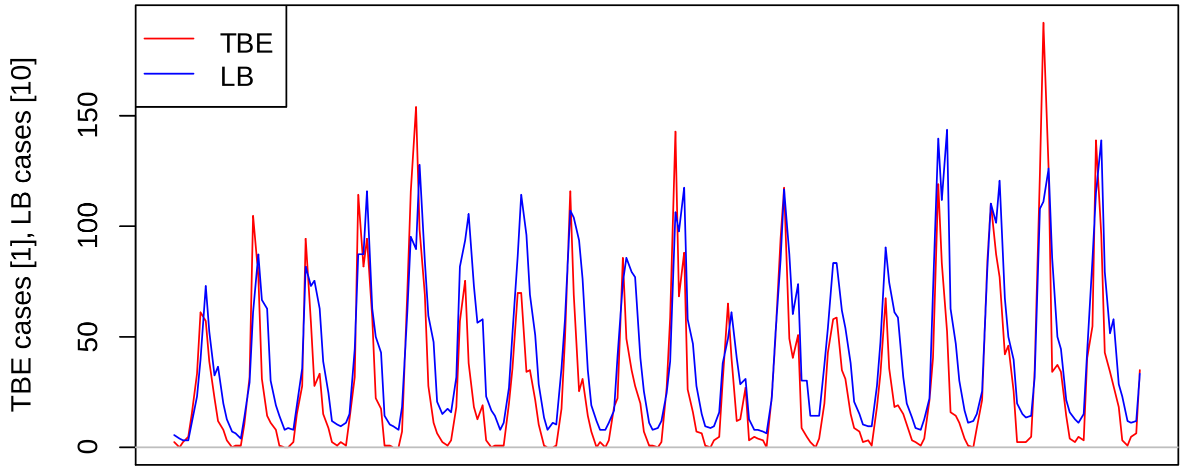

Figure S5: Seasonal patterns of reported TBE and LB cases. RKI data.
